# Differential Circular RNA Expression Profiles Following Spinal Cord Injury in Rats: A Temporal and Experimental Analysis

**DOI:** 10.3389/fnins.2019.01303

**Published:** 2019-12-10

**Authors:** Ronghua Wu, Susu Mao, Yaxian Wang, Shuoshuo Zhou, Yan Liu, Mei Liu, Xiaosong Gu, Bin Yu

**Affiliations:** ^1^School of Biology and Basic Medical Sciences, Soochow University, Suzhou, China; ^2^Key Laboratory of Neuroregeneration of Jiangsu and Ministry of Education, Co-innovation Center of Neuroregeneration, Nantong University, Nantong, China

**Keywords:** circular RNAs, spinal cord injury, astrocytes, proliferation, migration

## Abstract

Spinal cord injury (SCI), one of the most severe types of neurological damage, results in persistent motor and sensory dysfunction and involves complex gene alterations. Circular RNAs (circRNAs) are a recently discovered class of regulatory molecules, and their roles in SCI still need to be addressed. This study comprehensively investigated circRNA alterations in rats across a set time course (days 0, 1, 3, 7, 14, 21, and 28) after hemisection SCI at the right T9 site. A total of 360 differentially expressed circRNAs were identified using RNA sequencing. From these, the functions of the exonic circRNA_01477 were further explored in cultured spinal cord astrocytes. Knockdown of circRNA_01477 significantly inhibited astrocyte proliferation and migration. The circRNA_01477/microRNAs (miRNA)/messenger RNA (mRNA) interaction network was visualized following microarray assay. Among the downregulated differentially expressed mRNAs, four of the seven validated genes were controlled by miRNA-423-5p. We then demonstrated that miRNA-423-5p is significantly upregulated after circRNA_01477 depletion. In summary, this study provides, for the first time, a systematic evaluation of circRNA alterations following SCI and an insight into the transcriptional regulation of the genes involved. It further reveals that circRNA_01477/miR-423-5p could be a key regulator involved in regulating the changeable regeneration environment that occurs during recovery from SCI.

## Introduction

Spinal cord injury (SCI) is one of the most severe types of neurological damage and often results in permanent dysfunction of movement and sensation, severely affecting quality of life (Friedli et al., 2015; Kjell and Olson, 2016; Tica and Didangelos, 2018). The nervous system is a delicate system, orchestrated by numerous genes, and SCI strongly impairs such a coordinated environment (Hara et al., 2017). Understanding the cellular and molecular mechanisms underlying the pathophysiological events that occur during SCI in a systematic and comprehensive manner, in particular those related to gene regulation, is critical for developing new treatment strategies.

Circular RNAs (circRNAs) are a novel class of endogenous non-coding RNAs and have recently been the subject of much research interest and activity. In contrast to linear RNAs (messenger RNAs [mRNAs]), microRNAs (miRNAs), which are characterized by 5′ cap and 3′ tail structures, circRNAs are characterized by a covalently closed-loop structure that is generated by back-splicing (Bagchi, 2018). An increasing number of studies support the notion that circRNAs orchestrate gene expression by acting as miRNA sponges, interacting with RNA binding proteins, modulating transcription, and thus affecting numerous diseases, including cancer, as well as cardiovascular and neurological diseases (Floris et al., 2017; Ji et al., 2018; Li et al., 2018; Zhong et al., 2018).

In one study, Zhou et al. (2018) reported differentially expressed circRNAs involved in the pathogenesis of sciatic nerve injury, and several other studies have found alterations in the expression of circRNAs after sciatic nerve injury (Cao et al., 2017; Zhou et al., 2017). Recently, two publications have extended investigations of circRNA alterations to the field of rat SCI research (Qin et al., 2018; Zhou et al., 2019). Both of these studies reported that a large number of circRNAs were differentially expressed in the contused SCI site, on the basis of analyses of a single time point after injury. Zhou et al., found 150 significantly differentially expressed circRNAs at 6 h after SCI related to acute injury events, and Qin et al. (2018) identified 1,676 circRNAs with differential expression at 3 days after SCI. These studies indicate that alterations in circRNA expression show unique patterns during the process of SCI. Given the fact that SCI is a complex process with different events occurring during different time windows, we established an SCI model using multiple time points to investigate circRNA alterations at 0, 1, 3, 7, 14, 21, and 28 days. To our knowledge, this is the first study to survey the temporal expression of circRNAs following SCI, with the aim of providing a comprehensive assessment of the circRNAs involved in this process.

## Materials and Methods

### Animals

In this study, 252 adult male Sprague Dawley rats weighing 240–260 g were obtained from the Animal Center of Nantong University. All experimental procedures followed the guidelines of the Nantong University Institutional Animal Care and Use Committee. Efforts were made to minimize the number of animals used and their suffering. Rats were divided into groups (0, 1, 3, 7, 14, 21, and 28 days and their corresponding sham groups at the different time points), with six rats in each group. The experiments were repeated three times.

### Spinal Cord Injury Procedure and Histological Analysis

The T9 lateral hemisection surgical procedure followed the protocol used in our previous study, with some modifications (Chen et al., 2018). Briefly, after anesthesia, hair removal, and disinfection with 70% alcohol, an incision was made in the dorsal midline skin. The subcutaneous tissue extending from T8 to T10, and the muscle and tissue overlying the spinal column, were bluntly dissected away to reveal the T9 laminae. The hemisection was then carefully performed at T9 on the right side with an ophthalmic iris knife. The muscle layers were sutured, and the skin was secured with wound clips. The sham operation group only received the vertebral laminectomies to expose the spinal cord according to the above steps, without the clipping. To ensure injury consistency, the same experienced surgeon performed all surgeries. Some animals were randomly selected for histological analysis to validate the injury model. After fixation using 4% paraformaldehyde (PFA), the spinal cord tissues were cryo-protected with 30% sucrose and processed using a cryostat (CM3050s, Leica Biosystems Inc, Buffalo Grove, IL, United States) (section thickness for the spinal cord, 30 μm). The sections were stained with Hoechst 33342 for 15 min. All samples were visualized under a DMi8 microscope (Leica Microsystems, Wetzlar, Germany).

### RNA Sequencing and Analysis

Animals were deeply anesthetized with a compound anesthetic containing pentobarbital sodium (0.35 ml/100 g) via intraperitoneal injection. The proximal tissues within 5 mm of the T9 injury site on the spinal cord were carefully collected, stored in liquid nitrogen, and subjected to RNA sequencing (including total RNA sequencing and circRNA sequencing), which was performed by Shanghai Oebiotech. Library preparation was performed according to the recommendations of the sequencer manufacturer. The libraries were sequenced on an Illumina HiSeq X Ten platform (Illumina, Inc., San Diego, CA, United States), and 150-bp paired-end reads were generated. Raw data (raw reads) in FASTQ format were first processed using the NGS QC Toolkit^1^. CircRNAs were identified using CIRI (v2.0.3)^2^, the mapping software was Burrows-Wheeler-Alignment (BWA)^3^ (Li and Durbin, 2010), and circRNA expression was computed as RPM (spliced reads per million mapped reads). Differential expression analysis was then performed using the DESeq R package^4^; circRNAs with fold change ≥2 and *P*-value < 0.05 were considered to be differentially expressed (Ju et al., 2018). All data from this study can be viewed in NODE^5^ by pasting the relevant accession number (OEP000369) into the text search box or at this website^6^.

### RNA Isolation, General PCR, and Quantitative PCR

Total RNA from tissues and cells were isolated using TRIzol (Life Technologies, Carlsbad, CA, United States). The Ambion PARIS system (AM1921; Invitrogen, Carlsbad, CA, United States) was used to isolate nuclear and cytoplasmic RNA. RNA was quantified using the NanoDrop ND-2000 (Thermo Fisher Scientific, Carlsbad, CA, United States), and RNA integrity was assessed using the Agilent Bioanalyzer 2100 (Agilent Technologies, Santa Clara, CA, United States). The divergent primers of circRNAs were designed with the “primer 3” software^7^ and synthesized by the Thermo Fisher Scientific company (Shanghai, China). *Gapdh* was used as the reference gene. All primer sequences are listed in Table 1. To confirm the characterization of circRNAs, 2 μg of total RNA was treated with 0.2 μl of RNase R (Epicentre, Madison, WI, United States) in a 10 μl reaction volume for 15 min at 37°C. The primers of rno-miRNA-423-5p were designed and synthesized by Suzhou GenePharma (Suzhou, China). The first-strand cDNA was synthesized using random hexamer primers, or miRNA specific primers and HiScript Reverse Transcriptase (Vazyme Biotech, Nanjing, China), followed by general PCR amplification. RT-qPCR was performed in a total volume of 20 μl, including 1 μl of cDNA, 10 μM of primers, and 10 μl of 2 × SYBR Green qPCR Master Mix (Vazyme Biotech, Nanjing, China). Quantification via the 2^–Δ^ ^Δ^ ^CT^ method was used to calculate fold changes in gene expression between the different groups.

**TABLE 1:** The sequences of primers and siRNAs used in this study.

**Name**	**Sense (5′–3′)**	**Antisense (5′–3′)**
circRNA_09109	attgcagtgctgaagggga	aactggtcaacagccaattattc
circRNA_29751	gtcttgctacgattgtccacc	cctgcaatgtgctgcctac
circRNA_01477	gaatgtcacaagcagatgagaga	ctttgcatcaagacttgtggg
circRNA_03612	tatgggcaggcagagaagc	ctgatgaagtctcggtagcca
circRNA_25033	aactttccgtattacagagaggc	tgtatttgggtcaaggagtgc
circRNA_25269	gcagctccagcttcagaaca	acactcccctccaaagacaga
circRNA_26782	acgagagccattctgcaaca	atgccacaatccagagacaagt
circRNA_32637	ccctttttggggtctgttca	tgctcctcttcatcgtcagttag
circRNA_29417	tccgtggtaactgctttgct	caacgaattttgggtccatg
circRNA_14930	ctgcaggttgaaccaggtgt	tctacaagtcgccccagtg
circRNA_17241	cagtcacagagacacacaccacc	ctttgatcgcttctctcggg
circRNA_30754	tgcccccagctttcttatt	gctcaatcctttgggaactaa
circRNA_02670	tcagaagatgaaagaaaggagga	ttcccaaaaagcctactatcatc
circRNA_12788	tgtcatcctcactgtcatagcc	agcaggccccgaatacat
circRNA_16470	catagcagcaatgtggtgagag	agttatgcttcctgtgtggttca
circRNA_17645	aaacatcagctcaggagggtg	acagtcacggacctgaggatc
circRNA_20058	ttcttcacactacaaaaggcact	tattatgttggtggatcctgttc
circRNA_25527	ttgctggtgggcctcttg	tacgcagaggctcggagac
circRNA_26858	ggataatggcccaggtgat	agaacaagatgaaccaacacctc
*Gapdh*	ccatcactgccactcagaagact	acattgggggtaggaacacg
circRNA_01477 siRNA	cgaggggcuacacuugccatt	uggcaaguguagccccucgtt
Control (Ctrl) siRNA	uucuccgaacgugucacgutt	acgugacacguucggagaatt
*IL6R*	gcgaggagtaaagcatgtgg	ttccgtactgatcctcgtgg
*Ccnd3*	cttcctggccttgattctgc	gcgatcatggatggagggta
*Cxcl12*	gtgcccttcagattgttgca	gccgcctttctcttcttctg
*Cxcl14*	ctacagcgacgtgaagaagc	ttctcgttccaggcgttgta
*Ntrk2*	cagcaacgacgatgactctg	tgagctggctgttggtgata
*Inhbb*	gactggatcattgcacccac	tagaactcagcttggtgggg
*Gcnt1*	tgtgtgcgtctttggagttg	agcgttctaaggtctccagg
*Zfp592*	aggaacactggttggacctg	tggttgttgcgaatgtgttt
U6 snRNA	cgcttcggcagcacatatac	ttcacgaatttgcgtgtcatc
rno-miR-423-5p	gcacagtgaggggcagaga	tatggttgttctcgactccttcac

### Primary Astrocyte Culture and Small Interfering RNA Transfection

Primary astrocytes from postnatal day 1 rat spinal cord were prepared as we previously described (Hu et al., 2017) and incubated at 37°C in a humidified atmosphere of 95% air and 5% CO_2_. When cells became confluent, the cultures were shaken at 150 rpm for 16 h for purification. Astrocyte purification was confirmed by glial fibrillary acidic protein (GFAP; Sigma-Aldrich, St. Louis, MO, United States) immunocytochemical staining, and astrocyte cultures were considered appropriate for use when they were 95% positive for GFAP. Purified astrocytes at passage 2 were used for small interfering RNA (siRNA) transfection at a final concentration of 200 nM, using a NEPA21 electrical transfection instrument (Ishino et al., 2018). The siRNAs were synthesized by Suzhou GenePharma. Their sequences are listed in Table 1.

### Detection of Cell Proliferation and Migration

We used the 5-ethynyl-2′-deoxyuridine (EdU; RiboBio, Guangzhou, China) incorporation assay to test cell proliferation according to the protocol from our previous study (Zhou et al., 2014). At 24 h post-transfection, the astrocytes were digested and counted, and 5 × 10^4^ astrocytes were plated onto 0.01% poly-L-lysine-coated 24-well plates. At the indicated time points, 50 mM of EdU was applied to the cells, which were then incubated for an additional 2 h. After being fixed, the cells were analyzed using a Cell-Light EdU DNA Cell Proliferation Kit (RiboBio, Guangzhou, China). Cell proliferation was expressed as the ratio of EdU-positive cells to total cells, which was determined using images of randomly selected fields obtained on a DMi8 fluorescence microscope. In addition, we used cell counting kit-8 (CCK-8; Vazyme Biotech, Nanjing, China) to test cell viability according to the manufacturer’s protocol. Twenty-four hours after transfection, the astrocytes were digested, counted, and plated into 96-well plates. CCK-8 (10 μl) was added at the indicated time points, and the plates were incubated for an additional 2 h. Absorbance at 450 nm was measured to determine cell viability.

For the transwell migration assay, astrocytes were examined using 6.5-mm transwell chambers with 8-μm pores (Costar, Cambridge, MA, United States). The 700 μl complete medium was added into the lower chambers, and a 200 μl sample of Dulbecco’s modified Eagle medium (DMEM)/F12 containing 5 × 10^4^ resuspended astrocytes was transferred to the top chamber, where the cells were allowed to migrate at 37°C in 5% CO_2_. At the specified times, the upper surface of each membrane was cleaned with a cotton swab. Cells adhering to the bottom surface of each membrane were stained with 0.1% crystal violet (Beyotime, Nantong, China), imaged, and counted using a DMi8 inverted microscope. For the wound healing assay, 5 × 10^4^ astrocytes were seeded into the culture insert (Ibidi, Martinsried, Germany) in DMEM/F12 supplemented with 0.5% fetal bovine serum and 0.15 mg/ml of mitomycin C (Sigma-Aldrich, St. Louis, MO, United States) and incubated for 12 h. Afterward, the insert was removed with tweezers, yielding a standardized wound of 500 μm. The dish was washed and subsequently imaged in the medium described above for 24 h. Closure of the wound was monitored and photographed at multiple sites, and representative images were captured.

### Microarray Analysis and Competing Endogenous RNA Network Construction

Total RNA was quantified using the NanoDrop ND-2000 (Thermo Fisher Scientific, Carlsbad, CA, United States), and RNA integrity was assessed using the Agilent Bioanalyzer 2100 (Agilent Technologies, Santa Clara, CA, United States). The sample labeling, microarray hybridization, and washing were performed based on the manufacturers’ standard protocols. Feature Extraction software (v10.7.1.1; Agilent Technologies) was used to analyze array images to obtain the raw data. GeneSpring (v13.1; Agilent Technologies) was employed to complete the basic analysis of the raw data. First, the raw data were normalized with the quantile algorithm. Probes that had at least 100% of the values in any one out of all the conditions were flagged “detected” and were chosen for further data analysis. Differentially expressed genes were then identified based on their fold-change values. The threshold set for upregulated and downregulated genes was a fold change ≥2.0. Two software programs, namely, Miranda^8^ and PITA^9^, were used to predict circRNA_01477 target miRNAs and downregulated genes. Then related pairs of competing endogenous RNAs (ceRNAs) with endogenous competitive binding were obtained, and the ceRNA network was constructed. In Figure 4F, the green circles represent downregulated mRNAs, the green circle with a yellow outer ring represents the downregulated circRNA, and the red rectangles represent miRNAs.

We used the FDR_bh algorithm to correct the *P*-value calculated by the Fisher exact test algorithm, based on a Python script written by the company Shanghai Oebiotech. The Gene Ontology (GO) data were obtained from gene2go on the National Center for Biotechnology Information (NCBI) website, and the Kyoto Encyclopedia of Genes and Genomes (KEGG) data were obtained from the official KEGG website. GO analysis was performed to annotate genes from cellular components, biological processes, and molecular functions. The enrichment score calculated as the negative logarithm of the *P*-value was used to determine the statistical significance of GO biological process clusters targeted by differentially expressed genes. KEGG pathway analysis was used to determine the involvement of target genes in different biological pathways. The statistical significance of pathway correlations was determined from the enrichment score.

### Statistical Analysis

All experimental data are expressed as the mean ± SE. GraphPad Prism 5.0 software (GraphPad Software, San Diego, CA, United States) was used for data analysis. Statistical significance was assessed by one-way analysis of variance (ANOVA), and Student’s *t*-test was used to determine the significance of the differences between groups. Statistical results with *P* < 0.05 were considered to be statistically significant.

## Results

### Overview of Circular RNA Profiles During Spinal Cord Injury

**FIGURE 1:**
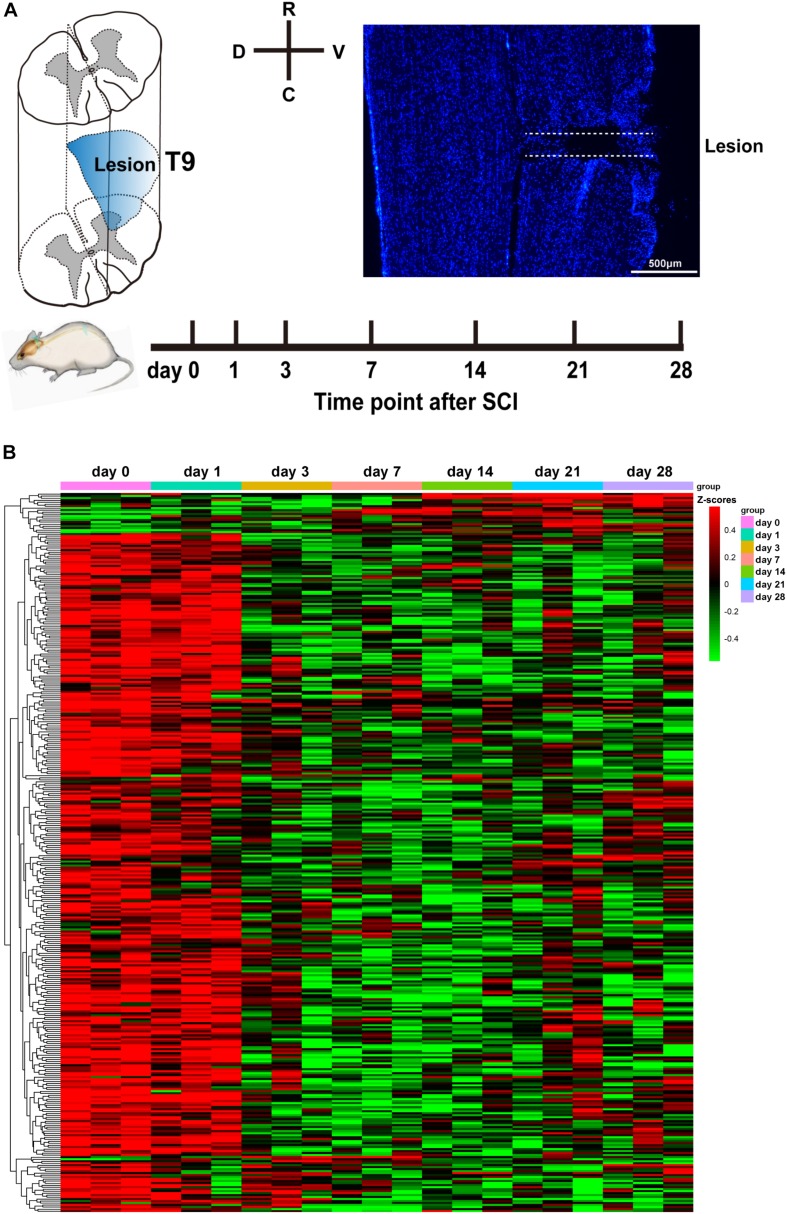
Analysis of differentially expressed circular RNAs (circRNAs) after spinal cord injury (SCI). **(A)** The diagram of the injury site, the time course of the experiment, and a representative Hoechst immunostaining image of the injury site. **(B)** Heatmap of circRNA expression at days 0, 1, 3, 7, 14, 21, and 28 following SCI. Red and green denote high and low expression, respectively. Each circRNA is represented by a single row of colored boxes, and each sample is represented by a single column.

To comprehensively and accurately investigate the temporal expression of circRNAs following SCI, we chose to perform lateral hemisection at T9 of the spinal cord in rats. According to the literature, hemisection is highly repeatable operation compared with the crush model of SCI. Figure 1A shows a diagram of the injury site, the experimental time course, and a representative immunostaining image of the injury site. After surgery, at 0, 1, 3, 7, 14, 21, and 28 days post-SCI, we collected spinal cord tissues within 5 mm of the injury site and stored them in liquid nitrogen. Then, we applied high-throughput sequencing to determine circRNA expression profiles using the principles described by Memczak et al. (2013). The results revealed that 360 circRNAs were differentially expressed at one or more of the time points (at days 1, 3, 7, 14, 21, or 28) after SCI (compared with the control day 0, *P* < 0.05; Figure 1B). Among these circRNAs, 31 were exonic, 33 intergenic, 1 intronic, and 295 sense-overlapping. It is of interest to note (Figure 1B) that the levels of 94% of the differentially expressed circRNAs decreased from day 3 onward. Based on existing knowledge of pathological processes during SCI, we know that the third day is a time point at which change occurs, because at 3 days, neutrophil infiltration and microglia activation are most conspicuous; and following this time point, these cells start to cause secondary damage (Fleming et al., 2006; Kjell and Olson, 2016). The differential expression of circRNAs occurring mostly at this time point may hint at the beginnings of later events. The expression trends of 360 circRNAs are listed in detail in Supplementary Table S1.

### Validation of Circular RNAs and Their Expression Patterns During Spinal Cord Injury

**FIGURE 2:**
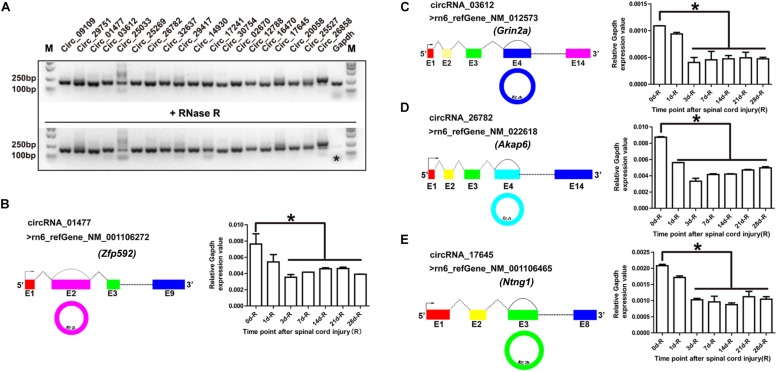
Validation of circular RNAs (circRNAs) and their expression patterns during spinal cord injury (SCI). **(A)** Agarose gel electrophoresis of the PCR products. Total RNA was treated with (lower panel) and without (upper panel) RNase R for 15 min at 37°C. Then the first-strand cDNA was synthesized using random hexamer primers, followed by general PCR. The image is color inverted, and the asterisk (^∗^) indicates *Gapdh* expression. **(B–E)** Schematic diagram of circRNAs and their expression patterns after SCI. The schematic diagrams of circRNA_01477 (**B**, left panel), circRNA_03612 (**C**, left panel), circRNA_26782 (**D**, left panel), and circRNA_17645 (**E**, left panel) show the RefSeq accession number of the reference genes and the circularized exon sites. The bar plots in the right panels show the statistical analysis of circRNA expression relative to *Gapdh* after SCI. Data are presented as the mean ± SE. ^∗^*P* < 0.05, Student’s *t*-test vs. day 0 (data were collected from injured proximal (R, rostral) spinal cord tissues at each time point).

Out of all the differentially expressed exonic circRNAs, 19 were validated by RNase R treatment and general PCR. As shown in Figure 2A, after RNase R treatment, only the *Gapdh* PCR product (shown by an asterisk) vanished, whereas the 19 circRNAs were still detectable. These 19 PCR products were identified by DNA sequencing, and the results demonstrated the characteristics of circRNA (shown in Supplementary Figure S1). On the basis of the DNA sequencing results, we identified the cyclization sites of their individual reference gene exons (shown in Figures 2B–E, left panel). To validate the RNA sequencing results, we selected circRNA_01477, circRNA_03612, circRNA_26782, and circRNA_17645, and confirmed their expression patterns using RT-qPCR. The results showed that the expression levels of the selected circRNAs were significantly decreased after SCI (shown in Figures 2B–E, right panel), and the expression patterns were consistent with the results of RNA sequencing.

### Effects of circRNA_01477 on Astrocyte Proliferation and Migration

**FIGURE 3:**
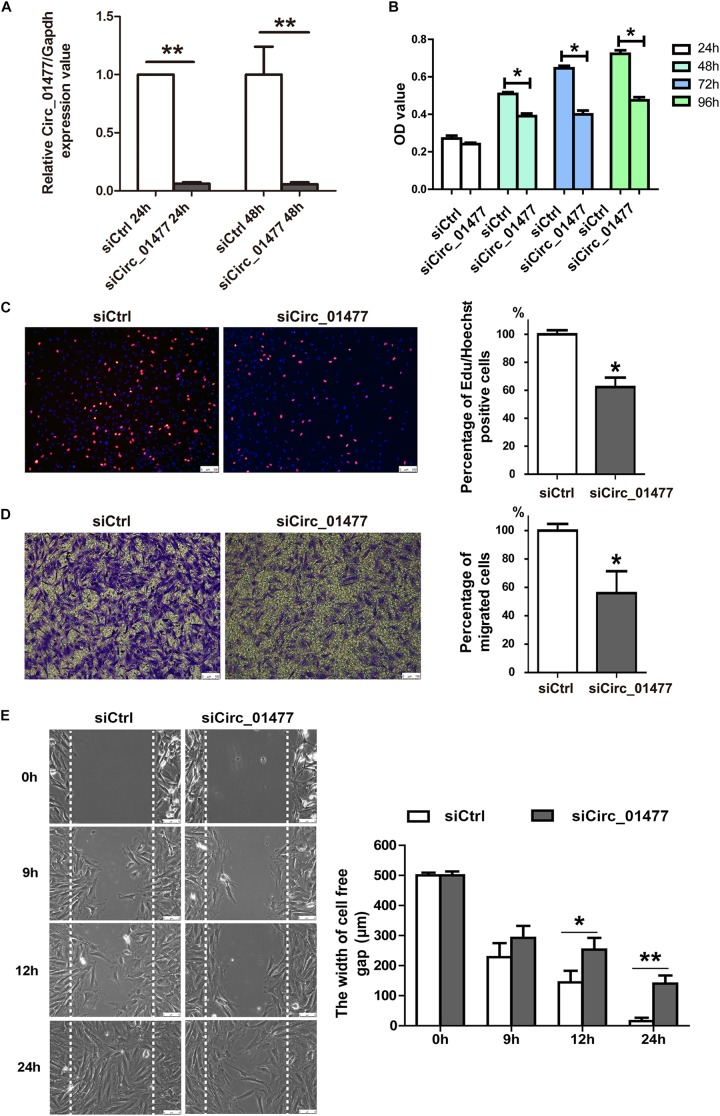
Effects of circRNA_01477 knockdown on astrocyte proliferation and migration. **(A)** Graph showing the silencing efficiency of the circRNA_01477 small interfering RNA (siRNA). After siRNA treatment for 24 and 48 h, relative levels of circRNA expression decreased by 93.8% and 94.3%, respectively. **(B)** The cell counting kit-8 (CCK-8) assay showed that astrocytic viability decreased by 23.3%, 38.1%, and 34.3% after circRNA_01477 siRNA treatment for 24, 48, and 96 h, respectively. **(C)** The 5-ethynyl-2′-deoxyuridine (EdU) assay showed that astrocytic proliferation decreased by 37.3% after circRNA_01477 siRNA treatment. Left panel: representative EdU images; right panel: statistical analysis. **(D)** The transwell assay showed that astrocytic migration decreased by 44% after circRNA_01477 siRNA treatment. Left panel, representative images; right panel, statistical analysis. **(E)** The Ibidi chamber assay showed that the 500-μm width of the cell-free gap decreased to 140.5 μm after circRNA_01477 siRNA treatment for 24 h and to 15.3 μm in the control (Ctrl) siRNA group. Left panel, representative images; right panel, statistical analysis. The data in panels **(A–E)** are presented as the mean ± SE, ^∗^*P* < 0.05 and ^∗∗^*P* < 0.01, Student’s *t*-test vs. control siRNA (*n* = 3).

Astrocytes are known to be the most abundant cells in the central nervous system, regulating neurotransmitters and maintaining neuronal function (Gaudet and Fonken, 2018). After SCI, astrocytes play crucial roles in SCI pathology by proliferating and migrating and by forming the glial scar that inhibits neural regeneration and plasticity (Anderson et al., 2016; Hara et al., 2017). Usually, at 7–14 days after SCI in rats, reactive astrocytes are already found clustering at the border of the injury (Kjell and Olson, 2016). We speculated that some of the differentially expressed circRNAs we had identified might contribute to astrocyte activation. Thus, we established proliferation and migration models of cultured astrocytes derived from rat spinal cords to investigate the effects of selected circRNAs, including circRNA_01477, circRNA_26782, and circRNA_17645. Because the expression of these circRNAs significantly decreased after SCI, we recreated this condition by knocking down circRNA expression in cultured astrocytes to clarify their effect on glial proliferation and migration. As shown in Figure 3, we found that circRNA_01477 knockdown (Figure 3A) significantly repressed astrocytic viability (Figure 3B) and proliferation (Figure 3C), whereas the other two circRNAs did not induce any obvious changes (data not shown). Furthermore, we investigated the effect of circRNA_01477 on cell migration and found that knockdown of this circRNA markedly inhibited astrocytic migration both in the transwell test (Figure 3D) and in the wound healing assay (Figure 3E). Meantime, we performed qPCR to measure the expression levels of circRNA_01477 and its host gene *Zfp592*. The results showed that at 1 and 3 days after SCI, expression of circRNA_01477 decreased significantly, whereas expression of its host gene *Zfp592* did not change markedly (shown in Supplementary Figure S2). On the basis of these results, we propose that circRNA _01477 may function as a regulator of glial proliferation and migration *in vivo*.

### Bioinformatic Analysis of circRNA_01477/MicroRNA/Messenger RNA Interaction and Validation of Their Interaction

To further elucidate the effects of circRNAs on target miRNA and mRNA interactions, we performed microarray analysis using knockdown of circRNA_01477 in cultured astrocytes from rat spinal cords. Compared with the control, a total of 1,528 differentially expressed mRNAs were identified resulting from circRNA_01477 knockdown, comprising 633 upregulated mRNAs and 895 downregulated mRNAs (Supplementary Table S2).

**FIGURE 4:**
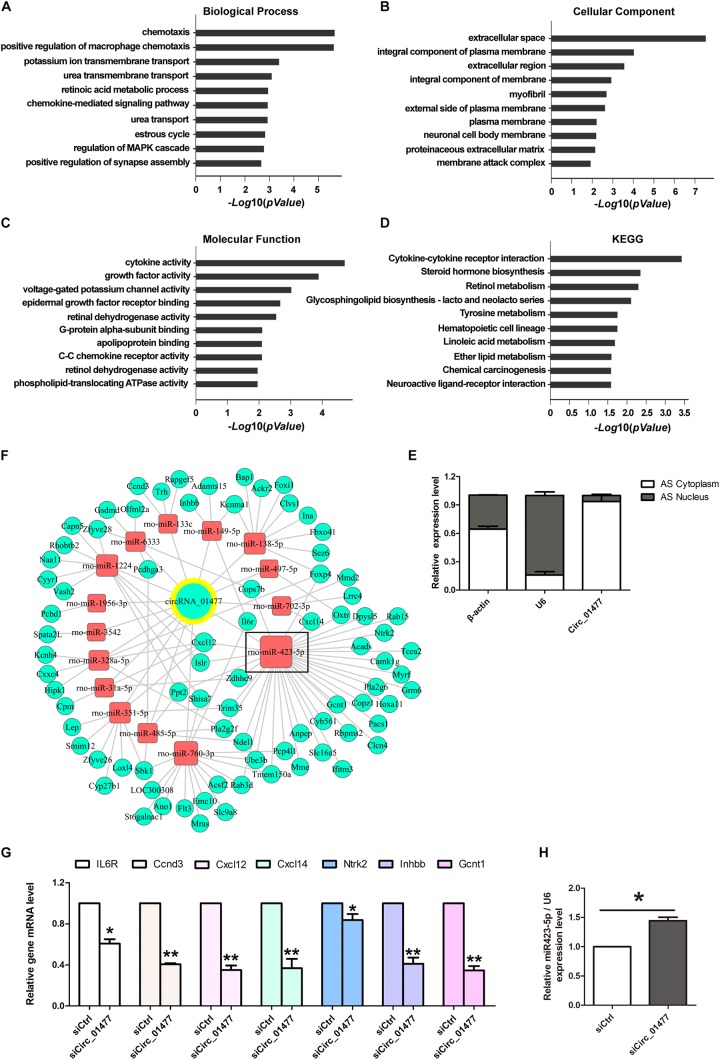
Bioinformatic analysis of circRNA_01477/microRNA (miRNA)/messenger RNA (mRNA) interaction and validation of the analysis. **(A–C)** Gene ontology (GO) analysis of target mRNAs regulated by circRNA_01477. The domains of “biological process” **(A)**, “cellular component” **(B)**, and “molecular function” **(C)** significantly changed their mRNA expression patterns after circRNA_01477 depletion in astrocytes. The enrichment score was calculated as -log_10_(*P*-value). **(D)** Kyoto encyclopedia of genes and genomes (KEGG) analysis. Potential signaling pathways involving the differentially expressed genes. The enrichment score was calculated as -log_10_(*P*-value). **(E)** Relative expression levels of circRNA_01477 in the cytoplasm and nucleus of astrocytes. **(F)** The competing endogenous RNA (ceRNA) network after circRNA_01477 knockdown, including 15 miRNAs and 81 mRNAs. Green circles represent downregulated mRNA, red rectangles represent miRNA, and the green circle with a yellow outer ring represents the downregulated circRNA. **(G)** Seven of these downregulated mRNAs were chosen for validation by qRT-PCR analysis, and the results were similar to those obtained from the microarray. **(H)** Relative expression level of rno-miRNA-423-5p after circRNA_01477 siRNA treatment in astrocytes. ^∗^*P* < 0.05 and ^∗∗^*P* < 0.01, Student’s *t*-test vs. control siRNA (*n* = 3).

We then performed GO and KEGG pathway analyses on siCircRNA_01477-induced differentially expressed downregulated mRNAs. The results indicated that the highest ranked clusters for each domain were as follows: chemotaxis in “biological process” (Figure 4A), extracellular space in “molecular component” (Figure 4B), and cytokine activity in “molecular function” (Figure 4C). The highest enrichment based on KEGG pathway analysis was for cytokine–cytokine receptor interaction (Figure 4D). The above informatics results suggest that circRNA_01477 may act as an important modulator for SCI-induced immuno-inflammatory responses, involving the related processes of cell proliferation and migration.

Meanwhile, using RT-qPCR followed by nucleic-cytoplasmic RNA isolation, we found that 93.5% of circRNA_01477 molecules are located in the cytoplasm, whereas the remaining 6.5% are in the nucleus (Figure 4E). We speculated that circRNA_01477 may play roles as a ceRNA and used two software packages, Miranda and PITA, to predict the targeting of miRNAs by siCircRNA_01477 and the corresponding downregulated genes. The related pairs of ceRNAs showing endogenous competitive binding were obtained, and the ceRNA network was constructed. As depicted in Figure 4F, 15 miRNAs and 81 mRNAs are included in the network surrounding circRNA_01477. As shown by this network, circRNA_01477 serves as an endogenous competitive RNA for 15 miRNAs, and these miRNAs could theoretically reduce expression of the 81 target genes.

We next chose to identify some related genes by RT-qPCR. The results (Figure 4G) showed that compared with the control, circRNA_01477 knockdown significantly decreased expression levels of the *IL6R*, *Ccnd3*, *Cxcl12*, *Cxcl14*, *Gcnt1*, *Inhbb1*, and *Ntrk2* genes. These results matched the expression pattern revealed by the GeneChip assay, which showed that expression of *IL6R*, *Ccnd3*, *Cxcl12*, *Cxcl14*, *Ntrk2*, *Inhbb*, and *Gcnt1* mRNA decreased by 12.93%, 9.96%, 6.65%, 7.84%, 12.45%, 9.80%, and 17.83%, respectively. These genes are usually involved in cytokine-induced cell proliferation and migration. Interestingly, we also found that four of these genes (*Cxcl12*, *Cxcl14*, *Gcnt1*, and *Ntrk2*) are predicted to be regulated by the same miRNA, namely, miR-423-5p. Therefore, we measured miR-423-5p expression after circRNA_01477 knockdown, and the result showed that inhibition of circRNA_01477 did indeed increase the level of miR-423-5p (Figure 4H).

## Discussion

The complexity of the pathophysiological processes resulting from SCI makes the development of effective therapies very challenging, because the extensive temporal changes in gene expression involved in the secondary phase of SCI are induced by vascular, cellular, and biochemical events (Liu et al., 2009; Yu and Gu, 2019). As important regulators of gene expression and molecular networks, the use of non-coding RNAs (ncRNAs) may serve as an innovative therapeutic strategy for treating SCI (Yu et al., 2015). ncRNAs are categorized into many different types, including small nuclear RNAs (snRNAs), small nucleolar RNAs (snoRNAs), transfer RNAs (tRNAs), ribosomal RNAs (rRNAs), PIWI (P-element-induced wimpy testis)-interacting RNAs (piRNAs), miRNAs, long ncRNAs (lncRNAs), and circRNAs. Previously, we found that the lncRNA TNXA-PS1 modulates Schwann cells (Yao et al., 2018), where miR-129 controls axonal regeneration during peripheral nerve injury (Zhu et al., 2018).

Recently, two publications have described the roles of circRNAs in SCI in rats (Qin et al., 2018; Zhou et al., 2019). Zhou et al., studied the alteration of differentially expressed circRNAs at just 6 h after SCI, whereas Qin et al. (2018) reported the expression patterns and potential functions of differentially expressed circRNAs at a single time window at 3 days after SCI. These studies have provided some information regarding the expression patterns, potential functions, and metabolic pathways of circRNAs. After SCI, a large number of astrocytes around the injury proliferate and undergo cell body hypertrophy, and cytoskeletal protein GFAP is significantly upregulated. Cells elongate and overlap to form a dense glial scar structure that surrounds the fibrous scar full of necrotic tissue and inflammatory cells (Burda and Sofroniew, 2014; Sofroniew, 2014). For many years, the glial scar has been regarded as a major obstacle to axonal regeneration and to the reestablishment of neural circuits. However, after central nervous system injury, glial scar tissue not only prevents the inflammatory response from spreading to the surrounding normal nerve tissue but also plays a role in repairing the damaged blood–brain barrier, stabilizing the local microenvironment and protecting the peripheral nerve tissue (Wanner et al., 2013; Anderson et al., 2016). These may suggest that astrocytes play different roles at different stages after SCI. Because SCI is a complex spatio-temporal process involving the interaction of injury-induced degeneration/regeneration, detailed investigative studies are worthwhile.

In this study, we presented data on differentially expressed circRNAs from seven time points within 28 days post-SCI to provide a comprehensive, spatio-temporal assessment of the circRNAs involved in this complex process. We established an SCI model by T9 lateral hemisection, and we collected circRNA data at days 0, 1, 3, 7, 14, 21, and 28 post-SCI to perform deep RNA sequencing. To ensure an accurate and repeatable operation, we followed the surgical protocol from our previous injury model (Liu et al., 2017; Chen et al., 2018), finally obtaining 360 circRNAs with significant differential expression in the spinal cord segment proximal to the injury.

We find it interesting to note from the 0–28 days’ heatmap (Figure 1C) that at 1 day post-SCI, the differentially expressed circRNAs showed a similar pattern to that at day 0. This result is similar to the report from Zhou et al., which found only 150 differentially expressed circRNAs at 6 h post-SCI (Zhou et al., 2019). Of the total of 360 differentially expressed circRNAs in this study, 94% of them decreased from day 3 onward. We suspect that this unexpected result may reveal a circRNA modulation checkpoint in the dramatically changing environment that occurs during the degeneration/regeneration transition. With reference to published reviews of pathological features in human or rat SCI (Fleming et al., 2006; Kjell and Olson, 2016; Yu and Gu, 2019), the switching events usually happen at 3–5 days’ post-SCI. At first 3 days, neutrophil infiltration and microglia activation prevail, and after 3 days, secondary damage comes to the fore (Bradbury and Burnside, 2019). With regard to the secondary stage, the formation of the glial scar mediated by astrocytic proliferation and migration is a main characteristic. Among these candidate circRNAs, we found that circRNA_01477 depletion in cultured astrocytes significantly inhibited astrocyte proliferation and migration. Based on the decreased expression pattern of circRNA_01477 after SCI, we suggest that circRNA_01477 downregulation may start to inhibit glial scar as an intrinsic spontaneous protection.

Circular RNAs in cytoplasm can function as ceRNAs for miRNAs, after which miRNAs usually act to inhibit the expression of their target mRNAs. After obtaining the mRNA expression profile of the circRNA_01477 knockdown, we constructed the network of circRNA_01477/miRNAs/mRNAs. Notably, these differentially expressed, downregulated mRNA molecules were clustered in the chemotaxis, extracellular space, and cytokine activity biological processes by GO analysis, and the cytokine–cytokine receptor interaction process in the KEGG pathway analysis. Chemotaxis is a phenomenon of cell-oriented movement in response to a chemical gradient in the extracellular environment (Shellard et al., 2018). Most of the screened genes identified regulate cell survival and proliferation and also contribute to cytoskeletal organization related to migration. Our results reveal that circRNA_01477 is probably a modulator involved in altering SCI-induced microenvironmental factors.

Among the downregulated differentially expressed mRNAs, four out of seven validated genes are controlled by miRNA-423-5p. We further identified miRNA-423-5p, which is significantly upregulated after circRNA_01477 knockdown. Previous studies have shown that upregulation of miRNA-423-5p significantly decreases cell proliferation and migration (Ge et al., 2018; Guo et al., 2018). In a very recently published study, Liu et al. demonstrated that promoting miR-423-5p expression reduces hydrogen peroxide-induced PC-12 cell injury (Liu et al., 2019). We speculate that miR-423-5p upregulation, which was induced by a reduction in the level of circRNA_01477 following hemisection SCI in rats, affects regeneration by inhibiting cell proliferation and migration.

In summary, this study provides a systematic evaluation of circRNA expression alterations following SCI, providing insights into the transcriptional regulation of the genes involved and revealing that circRNA_01477/miR-423-5p may be a key regulator of the changeable regeneration environment following SCI.

## Data Availability Statement

The datasets generated for this study can be found in NODE (http://www.biosino.org/node) by pasting the accession (OEP000369) into the text search box or through the URL: http://www.biosino.org/node/project/detail/OEP000369.

## Ethics Statement

The animal study was reviewed and approved by the Nantong University Institutional Animal Care and Use Committee.

## Author Contributions

RW, XG, and BY designed the study. RW, YW, and SZ performed the experiments. RW, SM, ML, YL, and BY analyzed or interpreted the data for the work. RW, ML, and BY composed the manuscript. All authors read and approved the final manuscript.

## Conflict of Interest

The authors declare that the research was conducted in the absence of any commercial or financial relationships that could be construed as a potential conflict of interest.

## Footnotes

1 http://www.nipgr.res.in/ngsqctoolkit.html

2 https://sourceforge.net/projects/ciri/files/CIRI2/

3 http://bio-bwa.sourceforge.net/

4 https://www.rdocumentation.org/packages/DESeq2/versions/1.12.3

5 http://www.biosino.org/node

6 http://www.biosino.org/node/project/detail/OEP000369

7 http://primer3.ut.ee/

8 http://miranda.org.uk/

9 https://genie.weizmann.ac.il/pubs/mir07/mir07_prediction.html
