# Supplementary material for: Differential Circular RNA Expression Profiles Following Spinal Cord Injury in Rats: A Temporal and Experimental Analysis
Source: Front Neurosci. 2019 Dec 10;13:1303. doi: 10.3389/fnins.2019.01303 (PMC6916439; doi:10.3389/fnins.2019.01303)
Supplement: Supplementary file 1 [file Data_Sheet_1.zip › 482683_Yu_SupMaterial-proof/Table 1-The sequencing results of the circRNA amplified PCR products..DOCX]

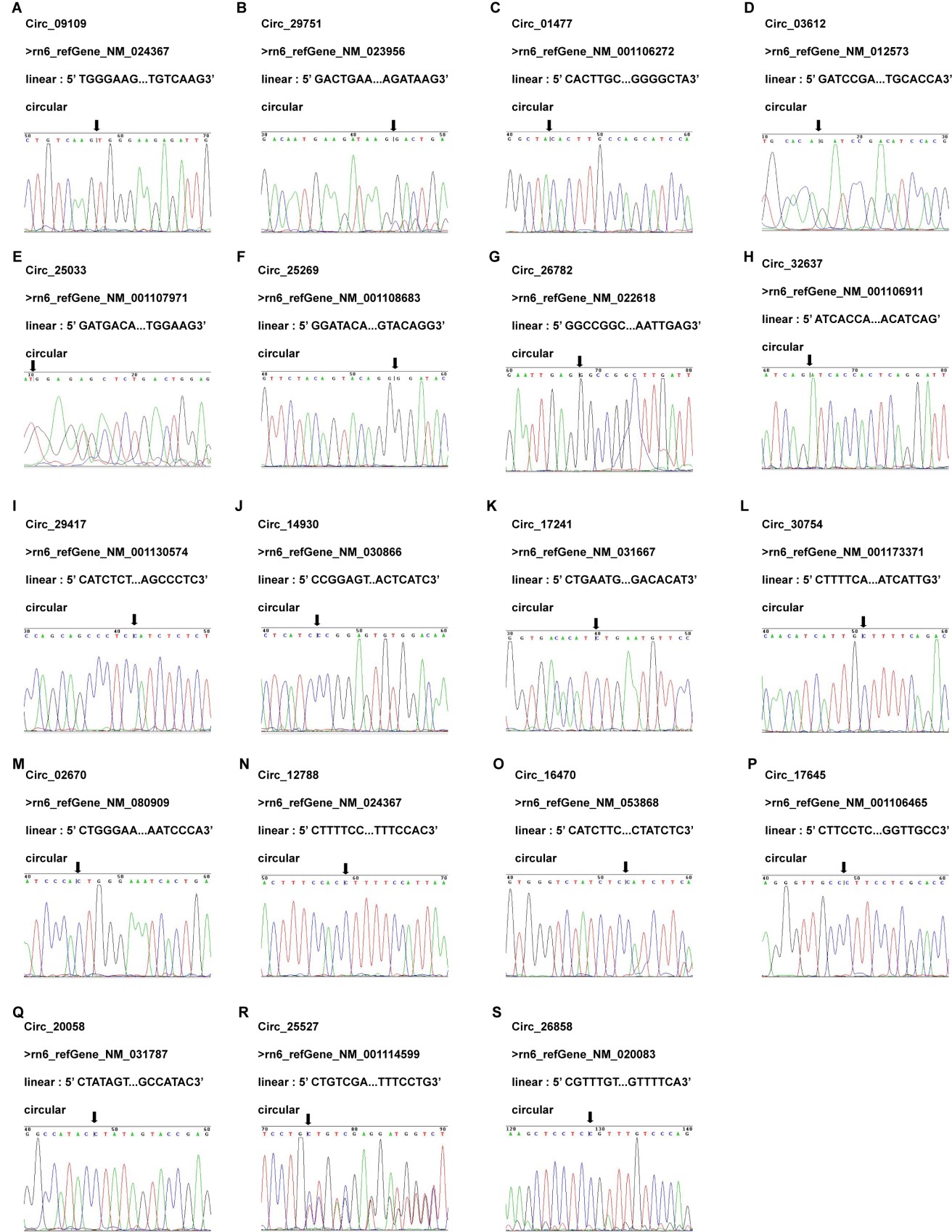


**Fig.S1.** The sequencing results of the circRNA amplified PCR products.

The RNase R treatment and PCR amplification were performed to 19 selected exonic circRNAs. Panels A-S showed the results of partial DNA sequencing, and the black down arrows showed circ_RNAs junctions.
