# Supplementary material for: Differential Circular RNA Expression Profiles Following Spinal Cord Injury in Rats: A Temporal and Experimental Analysis
Source: Front Neurosci. 2019 Dec 10;13:1303. doi: 10.3389/fnins.2019.01303 (PMC6916439; doi:10.3389/fnins.2019.01303)
Supplement: Supplementary file 1 [file Data_Sheet_1.zip › 482683_Yu_SupMaterial-proof/Table 4-The expression of circRNA_01477 and its host gene Zfp592 after SCI.DOCX]

**Fig.S2.** The expression of circRNA_01477 and its host gene *Zfp592* after SCI.

We performed qPCR to measure the expression levels of circRNA_01477 and its host gene *Zfp592*. The results showed that at 1 d and at 3 d after spinal cord injury, expression of circRNA_01477 decreased significantly, whereas expression of its host gene Zfp592 did not change markedly. Data are presented as the mean ± SE. * *P* < 0.05 and ** *P* < 0.01, Student’s t-test vs. day 0.
